# Risk Balancing of Cold Ischemic Time against Night Shift Surgery Possibly Reduces Rates of Reoperation and Perioperative Graft Loss

**DOI:** 10.1155/2017/5362704

**Published:** 2017-01-19

**Authors:** Nikos Emmanouilidis, Julius Boeckler, Bastian P. Ringe, Alexander Kaltenborn, Frank Lehner, Hans Friedrich Koch, Jürgen Klempnauer, Harald Schrem

**Affiliations:** ^1^General, Visceral and Transplant Surgery, Hannover Medical School, Hannover, Germany; ^2^Core Facility Quality Management & Health Technology Assessment in Transplantation, Integrated Research and Treatment Center Transplantation (IFB-Tx), Hannover Medical School, Hannover, Germany; ^3^Trauma and Orthopedic Surgery, Federal Armed Forces Hospital, Westerstede, Germany

## Abstract

*Background.* This retrospective cohort study evaluates the advantages of risk balancing between prolonged cold ischemic time (CIT) and late night surgery.* Methods.* 1262 deceased donor kidney transplantations were analyzed. Multivariable regression was used to determine odds ratios (ORs) for reoperation, graft loss, delayed graft function (DGF), and discharge on dialysis. CIT was categorized according to a forward stepwise pattern ≤1*h*/>1*h,* ≤2*h*/>2*h,* ≤3*h*/>3*h,*…, ≤*nh*/>*nh*. ORs for DGF were plotted against CIT and a nonlinear regression function with best *R*^2^ was identified. First and second derivative were then implemented into the curvature formula *k*(*x*) = *f*′′(*x*)/(1 + *f*′(*x*)^2^)^3/2^ to determine the point of highest CIT-mediated risk acceleration.* Results.* Surgery between 3 AM and 6 AM is an independent risk factor for reoperation and graft loss, whereas prolonged CIT is only relevant for DGF. CIT-mediated risk for DGF follows an exponential pattern *f*(*x*) = *A* · (1 + *k* · *e*^(*I* · *x*)^) with a cut-off for the highest risk increment at 23.5 hours.* Conclusions.* The risk of surgery at 3 AM–6 AM outweighs prolonged CIT when confined within 23.5 hours as determined by a new mathematical approach to calculate turning points of nonlinear time related risks. CIT is only relevant for the endpoint of DGF but had no impact on discharge on dialysis, reoperation, or graft loss.

## 1. Introduction

The standard procedure of kidney transplantation is a retroperitoneal graft-placement into the right or left* iliac fossa* with vascular end-to-side anastomosis of the donor artery to the recipient's iliac artery, an anastomosis of the donor vein to the recipient's iliac vein or vena cava, and a connection of the donor ureter to the recipient's bladder. The applied technique for ureter-to-bladder anastomosis [1–4] and the mode of vascular anastomosis (e.g., with or without aortic or venous patch) depend on the relations of the donor-to-recipient ureter/bladder anatomy and donor-to-recipient vascular three-dimensional geometry [5–9].

It is known that perioperative success or failure in kidney transplantation is related to donor organ quality [10–12], recipient comorbidities [13], quality of surgery [14, 15], and immunological parameters [16, 17] and that each of these categories contains hazards that can lead to one of the three major adverse events: (1) reoperation, (2) delayed graft function, or (3) graft loss. Furthermore, it is accepted that most of these hazards are unchangeable and elusive of control by the transplant surgeon at the time of scheduled surgery.

Nevertheless, there are two significant and time related hazards, cold ischemic time and* night shift* surgery, which are connected and sensitive to the timing of surgery. Therefore, it seems comprehensible that the transplanting surgeon might want to balance between these two variables, if CIT and* night shift* surgery would emerge as diametrically related hazards for the outcome of kidney transplantations.

## 2. Patients and Methods

### 2.1. Study Population

This is a single-center retrospective database analysis of all *N* = 1786 adult kidney transplantations performed between 1 January 2000 and 31 October 2013 at Hannover Medical School. All kidney transplants were performed after cold storage and a conventional cold perfusion during the organ harvest procedure using HTK preservation solution (Histidine-Tryptophan-Ketoglutarate, Custodiol® HTK, Dr. Franz Köhler Chemie GmbH, 64625 Bensheim, Germany). Machine perfusion techniques were not applied.

### 2.2. Inclusion and Exclusion Criteria

All consecutive standard kidney transplants defined as implantation of primary deceased single donor kidney transplantations into a pristine iliac fossa of an adult recipient (age > 18 years) were included. Living related transplantations, kidney retransplantations, and transplantations of organs with variant anatomy, such as double ureter or horseshoe kidneys, were excluded. Kidney transplants with synchronous procedures such as lung, heart, liver, or pancreas transplantation, ileum conduit, ureter-to-ureter anastomosis, nephrectomy, dialysis catheter removal, appendectomy, cholecystectomy, or inguinal hernia repair were excluded as well. After applying these inclusion and exclusion criteria a total number of *n* = 1262 patients with standard kidney transplants remained for analyses.

### 2.3. Data Collection

Data was collected on donor organs, recipients, surgery, and adverse events (Tables 1–6 and Supplemental Table in Supplementary Material available online at https://doi.org/10.1155/2017/5362704). Reasons for graft nephrectomy were discriminated into surgery related and nonsurgical causes by evaluation of the operative reports, clinical charts, and the pathological reports from the removed kidney transplants.

### 2.4. Study Endpoints

The investigated study endpoints were* delayed graft function [Yes/No]*, defined as temporary postoperative dialysis with dialysis-free hospital discharge,* hospital discharge on dialysis [Yes/No]*, early* postoperative graft loss [Yes/No]*,* postoperative graft loss due to surgical reasons [Yes/No]*, and* reoperation [Yes/No]* during the initial hospital stay.

### 2.5. Definition of Time Intervals for Day- and Nighttime Surgery

For the analysis of a possible risk development during day- or nighttime surgery the circadian 24 hours was analyzed using different permutations of defined time intervals with the goal of identifying time blocks of day- and nighttime surgery that are associated with the most significant risk increments for the investigated study endpoints. The start-times of surgery (skin incision times) determined the day or night shift intervals each kidney transplantation was assigned to.

### 2.6. Surgeons and Operative Procedures

Deceased donor kidney transplants were performed on 24 hours a day, seven days a week basis by a team of either two or three or seldom four surgeons. All ureter-to-bladder anastomoses were performed using the Gregoir-Lich antireflux technique [1]. Donor arteries were anastomosed end-to-side to the recipient's iliac artery. Performance of the operation with or without aortic patch as well as the choice of the exact anatomic site for anastomoses varied from case to case according to the individual vascular geometry of the recipient's and donor kidney's arteries. The same principle was applied for venous anastomoses to the iliac vein or vena cava.

### 2.7. Shift Regulations for Surgeons and Surgical Staff

**Figure 1:**
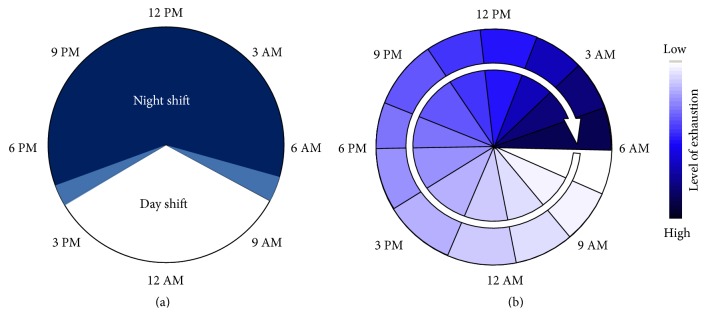
(a) Shown is the distribution of night- and daytime shifts over 24 hours at our institution. Regular working hours at our institution are 07:30 AM to 4:30 PM. Included are two hand-over periods of 30–45 minutes for each shift change* (light blue sectors)*. Only in cases of high urgent emergencies (e.g., bowl perforations, intraabdominal bleeding, and polytrauma situations) would surgery be started within those intervals. If arrival of an upcoming kidney transplantation is scheduled after 6 AM, the transplantation is usually planned to start with the day shift team after 7:30 AM. (b) This circle with the increasing dyeing of the sectors symbolizes the increasing levels of exhaustion that can be caused by sleep deprivation and a continuous workload over the course of a 24-hour shift.

Regular working hours at our institution are from 07:30 AM to 4:30 PM with a 30 minutes' rest-time. Surgeons and staff who are assigned for* night shift* will start working at 07:30 am and they have the same workload during the day as any other surgeons or staff members, who are not assigned for night shift.* Night shift* surgery then starts at 04:30 PM and ends at 07:30 AM the next morning (Figure 1(a)). Thus, surgeons or staff members who are assigned for* night shift*s do have an overall work-time of 24 hours (Figure 1(b)). After a continuous 24-hour shift all surgeons and staff are obliged to have 24 h off-time by law and will not start working again until the next morning. During* night shifts* there are no elective procedures. The night shift team consists of 3 surgeons: one senior surgeon, one senior resident, and one junior resident. Further, the* night shift* team also includes two examined scrub nurses. Surgeons, scrub nurses, and anesthesiologists are assigned randomly to night shifts without regard to upcoming kidney transplantations.

### 2.8. Surgical Experience and Teaching

A surgeons' experience with kidney transplantation was estimated by the number of performed transplants he accumulated until the date of each transplant (labeled here as CUSUM). This measure of surgical experience was examined for significant differences in distribution between investigated time intervals. Teaching transplants were defined as operations performed by a primary surgeon with less surgical seniority as compared to the assisting surgeon.

### 2.9. Statistical Analyses

#### 2.9.1. Missing Data

Patterns of missing data were analyzed by Little's test for Missing Data Completely at Random (MCAR) using SPSS Version 22 (PASW Statistics Inc., IBM, Somers, NY, USA). Missing data had a verified MCAR pattern, if significance level was *p* > 0.05.

#### 2.9.2. Uni- and Multivariable Regression Analysis

Binary univariable regression analysis was used to determine the odds ratios and the significance level of risk factors for the investigated study endpoints. Risk factors with significant *p* values < 0.05 as well as purposefully selected risk factors with *p* values < 0.200 were chosen for inclusion into risk-adjusted multivariable regression after exclusion of multicollinearity with the goal of determining significant independent risk factors for the study endpoints. *p* values < 0.05 were defined as significant. Chi^2^ tests and Levene's tests were performed with Minitab 17 (Minitab Inc., State College, Pennsylvania, USA). Univariable and multivariable regression statistics were performed with SPSS Version 22 (PASW Statistics Inc., IBM, Somers, NY, USA).

#### 2.9.3. Conversion of the Continuous Variable of Cold Ischemic Time to a Categorical Variable and Modelling of a Nonlinear Regression Function of the CIT Dependent Risk for Delayed Graft Function

As a result of the multivariable regression analysis of the investigated study endpoints we found that cold ischemic time (CIT) was only relevant for the study endpoint of* delayed graft function (DGF) (see *Results*).* In order to investigate the relationship of CIT with the binary endpoint of* DGF [Yes/No]* we calculated the risk increments for DGF as odds ratios (OR) for each additional hour of cold ischemic time (CIT). In order to do so we categorized the continuous variable CIT in a stepwise forward fashion according to the pattern ≤1*h*/>1*h,* ≤2*h*/>2*h,* ≤3*h*/>3*h,*…, ≤*nh*/>*nh*, and so forth. The calculated ORs for each hour of CIT progression were then graphically plotted as a function of OR by CIT to visualize the pattern of risk development over time. The approach of an hourly stepwise forward calculation of risk increments and the avoidance of the traditional technique of linear regression modelling enabled circumventing the otherwise determined (analysis inherent) result of a linear correlation between DGF risk and CIT. Thus, neither a nonlinear function nor a linear function of DGF by CIT was excluded from the beginning. Contrary to the traditional approach of an intentional choice of the analysis method we let the plotted data guide our choice for a rightful regression modelling to find a mathematical function with best estimated fit (lowest SSE and best *R*^2^).

**Figure 2:**
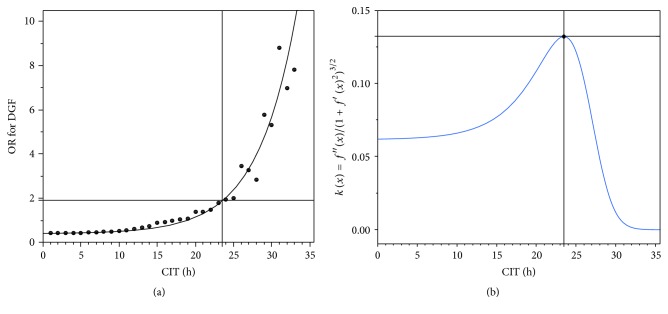
(a) Increments of odds ratios (OR) for* delayed graft function* were calculated and plotted per increment of CIT-hours. A mechanistic asymptotic regression function *f*(*x*) = *A* · (1 + *k* · *e*^(*I* · *x*)^) (*A* = asymptotic, *k* = scale, and *I* = increment) with best estimated fit was chosen for further calculations. The function *f*(*x*) = 0.4175662 · (1 + 0,035169 · *e*^(0.196467 · *x*)^)* (black line)* was finally identified with lowest SSE = 10.3141 and best *R*^2^ = 0.94 (a). (b) The point of highest acceleration in risk increment was then calculated by insertion of the regression equation *f*(*x*) = 0.4175662 · (1 + 0,035169 · *e*^(0.196467 · *x*)^) into the curvature formula *k*(*x*) = *f*′′(*x*)/(1 + *f*′(*x*)^2^)^3/2^ with *k*_max._ = 0.13 to 23.5 hours at an odds ratio of 1.9 (cross hairs at* black* (a) and blue lines (b)). The calculated CIT cut-off of 23.5 h was then used in univariable regression analysis to calculate the risk of delayed graft function development for CIT > 23.5 h to a hazard ratio of 3.713 (CI 2.215–6.225; *p* < 0.001).

Using JMP® Pro Version 11.2.0 (SAS Institute Inc., Cary, NC, USA) we finally identified a nonlinear regression function of the type *f*(*x*) = *A∗*(1 + *k* · *e*^(*I* · *x*)^) (*A* = asymptotic, *k* = scale, and *I* = increment) with lowest SSE and best *R*^2^ (Figure 2(a)).

First and second derivate of that function were implemented to the two-dimensional* curvature formula* [27] *k*(*x*) = *f*′′(*x*)/(1 + *f*′(*x*)^2^)^3/2^. The first derivate of the curvature formula with *k*′(*x*) = 0 was then used to calculate the point of highest acceleration in risk development and defined as CIT cut-off for risk balancing (Figure 2(b)).

## 3. Results

### 3.1. Missing Data

Missing value (MV) analysis by Little's MCAR verified a pattern of data completely missing at random with *p* values ranging from 0.434 to 0.795 (Tables 3, 4, 5, 6, and 7). There was no necessity for imputation of missing data.

### 3.2. Time Intervals for Day- and Nighttime Surgery

After several permutations (data not shown) the following 3-hour interval division of 24 h was identified as the time blocks that provide the best resolution with the highest significance levels and the highest calculable hazard ratios for the investigated study endpoints: 12 PM–3 AM, 3 AM–6 AM, 6 AM–9 AM, 9 AM–12 AM, 12 AM–3 PM, 3 PM–6 PM, 6 PM–9 PM, and 9 PM–12 PM (Table 1).

### 3.3. Surgical Experience and Teaching Operations during Daytime Shifts and Night Shifts

There were no significant differences between the frequencies of individual surgeons' postoperative complications that caused subsequent reoperations in each investigated three-hour interval (*p* < 0.05, Chi^2^). The proportion of teaching operations during the critical hour from 3 AM to 6 AM was different neither to the extreme with lowest (9 PM–12 AM) nor to the extreme with highest (6 AM–9 AM) proportion of teaching operations (*p* > 0.05, Chi^2^ test) (Table 2, Supplemental Figure a). The variances of surgical experience as expressed in calculated CUSUM of the operating primary surgeons between all 3 h intervals were not significantly different (*p* = 0.627, Levene's test) (Table 2, Supplemental Figure b). The 1st surgeons CUSUM had no significant impact on any of the investigated adverse events (Tables 3, 4, 5, 6, and 7).

### 3.4. Circadian Daytime Related Risk Development

In the next step we analyzed how big the risk was for any of the binary endpoints within each of the 3 h daytime intervals. Univariable binary regression analysis showed a substantial increase in the risk for* reoperation*,* perioperative graft loss*, and* perioperative graft loss due to surgical reasons* for the 3 AM to 6 AM time interval. In contrast we observed a reduction in risk of* delayed graft function* for the 3 AM to 6 AM* night shift* interval, although this tendency was statistically not significant (Table 1).

### 3.5. Independent Risk Factors for Reoperation

Risk-adjusted multivariable regression analyses revealed that donor age, donor BMI, and* night shift* surgery from 3 AM to 6 AM were significant independent risk factors for reoperation during the initial hospital stay. Stenting of ureter anastomosis on the other hand significantly reduced the risk of reoperation (Table 3).

### 3.6. Independent Risk Factors for Perioperative Graft Loss

Donor age,* night shift* from 3 AM to 6 AM, and recipient BMI were independent significant risk factors for perioperative graft loss (Table 4). More than one arterial anastomosis was a significant hazard in the univariate regression but did not reach significance (*p* = 0.069) in the multivariate regression analysis.

### 3.7. Independent Risk Factors for Perioperative Graft Loss due to Surgical Reasons

*Night shift* between 3 AM and 6 AM, recipient BMI, and more than one arterial anastomosis were identified as independent significant risk factors for perioperative graft loss due to surgical reasons (Table 5).

### 3.8. Independent Risk Factors Risk Factors for Delayed Graft Function

Donor age, recipient BMI, and cold ischemic time were significant independent risk factors for delayed graft function (Table 6).

### 3.9. Independent Risk Factors for Hospital Discharge on Dialysis

Donor age was the only independent significant risk factor for hospital discharge on hemodialysis (Table 7).

### 3.10. Modelling of the Nonlinear Regression Function of the CIT Dependent Risk for Delayed Graft Function and Calculation of the Cut-Off Point of Highest Risk Increment

Multivariable regression analyses revealed that CIT was relevant only for the endpoint of* delayed graft function*. The calculated odds ratios (OR) for* delayed graft function* per CIT-hour were plotted and a nonlinear regression function with best estimated fit was chosen. The resulting mechanistic asymptotic regression function *f*(*x*) = 0.4175662 · (1 + 0,035169 · *e*^(0.196467 · *x*)^) (Figure 2(a)) with smallest SSE fit (*R*^2^ = 0.94) was then implemented into the two-dimensional curvature formula [27] *k*(*x*) = *f*′′(*x*)/(1 + *f*′(*x*)^2^)^3/2^ for the calculation of highest risk acceleration. The highest risk acceleration (*k*_max._: the point at which the risk increment changes the quickest) was then identified using the first derivate *k*′ of that risk acceleration function at 23.5 hours (Figure 2(b), *k*_max._ = 0.13) and was defined as the* CIT cut-off* for* delayed graft function*. The parameter estimates for 23 hours and 24 hours were 0.578 (OR 1.783; CI-95%: 1.07–2.97) and 0.661 (OR 1.937; CI-95%: 1.06–3.542), respectively. All kidney transplantations were then categorized into belonging to the group either below or above the calculated CIT cut-off of 23.5 h. The new variable* above CIT cut-off* [yes/no] was then included into a binary regression analysis and the hazard ratio for* delayed graft function* beyond 23.5 h was calculated to OR = 3.713 (CI 2.215–6.225).

## 4. Discussion

This study reports for the first time that transplantation in the early morning hours between 3 AM and 6 AM is an independent significant risk factor for early outcome after kidney transplantation (Tables 1 and 3–7). This study is also an in-depth analysis about the significance of cold ischemic time (CIT) for the outcome of kidney transplantation and about the methodology of how to calculate risks that are mediated by time related variables such as CIT. We show that risk balancing between nighttime kidney transplantation and cold ischemic time is always necessary, even if this violates the holy grail of CIT reduction.

Variables that reflect the surgical complexity such as the number of renal arteries and veins [14, 28, 29], length of ureter and blood vessels [6], recipient BMI [30–32], and recipient age [33] as well as immunological parameters [16, 17] and patient comorbidities [13] cannot be changed by the operating surgeon on the day of surgery. As soon as a donor kidney is allocated these preconditions are unchangeable. CIT and the daytime of transplantation on the other hand can be influenced by the operating surgeon to some extent. Therefore, it is interesting to note that the variables CIT and the onset time of a kidney transplantation between 3 AM and 6 AM are competing hazards for perioperative outcome.

Despite the small number of patients who underwent transplanting within the 3 AM to 6 AM interval it must be noted that initiation of transplantation during that interval significantly increased the risk of* reoperation, perioperative graft loss*, and* graft loss due to surgical reasons* (Tables 1 and 3–5), while strikingly the relevance of prolonged CIT was negligible for all the studied endpoints, except the endpoint of delayed graft function (DGF), for which we identified a cut-off at 23.5 hours of CIT (Figure 2). This result is in line with the previously published findings of the Collaborative Transplant Study (CTS), which reported on more than 60,000 cases that graft survival was only marginally influenced by ischemia times up to 24 hours [24].

When putting these two findings together the logical consequence is to avoid transplantations between 3 AM and 6 AM in the morning, at least as long as CIT is not prolonged above the turning point of 23.5 hours. Following this approach would replace the so far undisputed transplant dogma of* CIT reduction by any means* with a strategy of finding a meaningful balance between two possibly competing hazards, CIT and daytime of surgery.

Not only is it a judgment of common sense that working at late night hours inevitably induces increased error and defect rates, but also it has been shown extensively that sleep deprivation and mental fatigue negatively impact on key cognitive functions such as attention [34–37], working memory [38, 39], risk assessment [40], and decision making [41]. Thus it is no surprise that there are a number of studies that found that surgery during night shifts is hazardous and potentially deteriorates quality and outcome in comparison to daytime surgery [42–44]. However, numerous studies reported that day and night shift surgery are not different with respect to outcome [45–47]. The explanation of these contradictory results is reasonably simple, at least at face value. All of these latter studies defined* night shift* surgery under inclusion of evening hours (*e.g.,* 8 PM–8 AM [48], 4 PM–6 AM [44], and 3 PM–3 AM [43]) and failed to scope at those hours of the night between 12 PM and 6 AM when the circadian rhythm most persuasively demands sleeping [49]. In this context it must be noted that conversion of continuous variables such as daytime [hh:mm:ss] into a binary format (e.g., 8 PM–6 AM versus 7 AM–7 PM) introduces a significant loss of resolution. As a consequence, possible risk increments are easily overseen. In order to meet that concern and to enhance the contrast of the investigated daytime variable it is important to set the boundaries for binary conversion not arbitrarily but with respect to plausible causation. For this study we have investigated several permutations of different day- and nighttime blocks. These investigations showed that the finally chosen 3-hour intervals were identified as time blocks that provide the best resolution with the highest significance levels and the highest calculable hazard ratios for the investigated study endpoints (Table 1). This approach resulted in the finding that the time interval between 3 AM and 6 AM was associated with significantly increased complications after kidney transplantation, which is a plausible result with respect to a surgeons' fatigue caused by sleep deprivation and the fact that humans are not nocturnal mammals and do not express the typical phenotypes of nocturnal animals. Therefore, the* night shift* hours from 12 PM to 6 AM can be intuitively understood as a hazard in comparison to day-light surgery. Analyzing deteriorated outcomes and a possible causation by* night shift* surgery though requires setting the boundaries of* night shift* hours in a way so that the time interval likely correlates with sleep deprivation and laps of circadian rhythm.

The surgeon usually has to organize and schedule the transplantation procedure. This involves informing involved personnel (intensive care unit, anesthesiologist, scrub nurses, and surgical team), carrying out the recipient examination and repetitive communication with the transplant coordinator. Further, the donor organ needs to be inspected and prepared ahead of the actual start of anesthesia. Consequently, the transplant surgeon usually is up on his feet at least one hour before the actual start of the operating procedure. The critical time period of 3 AM to 6 AM, which reflects the start-time of the operation, usually requires a wake-up call at least 1-2 hours earlier. Moreover, if the transplant center has no specialized transplant team in stand-by, who could be called in to perform an organ transplantation at any time, then the in-house on-call surgeons, who are often involved in other emergency procedures and consultations, will have to perform the transplantation in between all other emergency procedures, as is the case in our center. This implies that the on-call surgeons frequently have no time to rest before the onset of the transplant procedure, which possibly explains the high risk increment during the last hours of a 24-hour night shift. There were no differences in neither age, seniority, experience, nor training, when comparing daytime shifts and night shifts. Respectively, there were no differences in seniority and transplant surgery experience: for neither the surgeons, nor anesthesiologists, nor nursing staff or any other caregivers. This may be different from institution to institution and may thus lead to different results.

Theoretically there is a possible impact of surgical support staff's fatigue on surgical outcome as well, because the surgical support staff has similar day- and nighttime shifts. But the staff is not responsible for and not involved in the management process of emergencies, such as interdisciplinary telephone conferences, ER visits, patient examinations, discussion of CT scan results, and other time consuming nightly events, which do keep the surgeons from sleeping. Because the staff is not involved in processing of nonsurgical emergencies and because the staff of the general/transplant surgery department is exclusively assigned to the general/transplant surgery team, their workload during night shifts is significantly less.

Another possible influence might be the experience level of the supporting surgical staff. But the staff's team always consisted of one senior scrub nurse and one learning scrub nurse, who were randomly assigned to the surgeons' night shift team. There was no systematic hazardous team-bias that possibly could have altered the quality of surgery during night shifts or certain night shift hours.

Fechner et al. [48], even though they did not investigate surgery related complications of the primary hospitalization period when most surgery related complications usually unfold, found that* night shift* surgery prevailed as a significant hazard in their analysis and concluded that* night shift* surgery with the goal of reducing CIT at any costs might not be a wise decision.

In our current analysis* delayed graft function* significantly correlated with CIT prolongation (Table 6), while in contrast the period from 3 AM to 6 AM was advantageous. This observation is plausible, apparently due to the fact that CIT of kidneys transplanted between 3 AM and 6 AM was significantly shorter as compared to kidneys that had been transplanted outside the 3 AM–6 AM time interval (data not shown). Furthermore, CIT did not stratify as a major risk for* discharge on dialysis* (Table 7) and was neither a significant independent risk factor for* reoperation* (Table 3), perioperative graft loss (Table 4), nor a risk for* graft loss due to identified surgical reasons* (Table 5).

We found that more than one arterial anastomosis was a significant risk for* perioperative graft loss due to surgical reasons* (Table 5), while the number of arterial anastomoses did not stratify as a hazard for perioperative graft loss in general (Table 4). We also confirm the findings of others [30, 31] that recipient obesity is a risk for* delayed graft function* and surgical complications. However, obesity was no independent significant risk factor for* discharge on dialysis* (Table 7).

One consequence of intentional shifting of the start-time of a transplant operation from the 3 AM–6 AM interval to a day shift interval after 6 AM is the likely collisions with scheduled subsequent elective surgeries. Our data though justifies the postponing of the elective surgery schedule in order to avoid likely higher complication rates of night shift kidney transplantations as in our opinion these aspects outweigh the negative consequences of a delayed elective surgeries schedule, because higher rates of complications not only affect each transplanted patient, but also have substantial negative economic consequences for the hospital as well.

Pulsatile perfusion preservation could be a means to avoid delayed graft function caused by prolonged CIT [50] and may be a valuable tool if CIT prolongation past the identified cut-off of 23.5 hours might be unavoidable.

The conventional approach to odds ratio calculations for CIT-associated risks usually is a comparison of a predefined CIT interval against the mean risk that lies outside this predefined interval (two-sided; before and after) [18–20, 22–26, 51] (Table 8). This conventional approach ignores the fact that CIT is a continuous and linear progressive variable and that any risk which is dependent from cold ischemia is a function of time also. But for the calculation of a risk development as a function of any continuous variable it is necessary to calculate the risk increments for each step of CIT category progression. And this is only possible if CIT is converted into a categorical variable in a cumulative stepwise forward fashion. But to our knowledge there are no published studies that have utilized such a stepwise statistical analysis of CIT-minutes or CIT-hours against any study endpoint. All published statistical analysis used a blockwise two-sided risk calculation [18–20, 22–26, 51, 52] (Table 8).

Furthermore, when converting a continuous variable to a categorical variable it is necessary to generate categorical steps that are small enough. Otherwise, significant risk increments or any nonlinear risk development is artificially concealed due to lack of resolution. Most published analyses though are based upon CIT categorizations with resolutions ranging from 6 to 12 hours [18, 19, 23–26]. In some studies CIT was even divided only in a binary fashion [53, 54]. Even though some authors have claimed to have shown that each additional hour of CIT significantly increases the risk for graft failure [18], none of them actually made use of 1-hour CIT intervals to calculate the risk increments.

## 5. Conclusion

This study demonstrates a new mathematical method for calculating the cut-off value for the largest CIT-mediated risk increment for adverse early outcomes such as delayed graft function. The proposed method for calculating time related risk increments and cut-offs utilizes a cumulative stepwise forward categorization of CIT. We believe that this approach is appropriate when the mathematical relation between a continuous variable such as time and the odds ratio for an adverse event is unknown. In detail, this method allowed the deduction of a nonlinear regression function with the highest SSE and *R*^2^ value to describe the relation of CIT on the *x*-axis with the stepwise calculated odds ratios for DGF on the *y*-axis (Figure 2(a)).

Furthermore, we demonstrate that utilizing the* curvature formula* [27] *k*(*x*) = *f*′′(*x*)/(1 + *f*′(*x*)^2^)^3/2^ in combination with nonlinear modelled regression equation is an elegant method to determine cut-offs and turning points of time related risks. We believe that this methodology is a novel approach and is of general relevance. The clinically relevant conclusions of this study are to avoid kidney transplantation between 3 AM and 6 AM in the morning in order to improve overall outcome, as long as prolongation of CIT is confined within 23.5 hours and that CIT is only relevant for the endpoint of* delayed graft function* but had no impact on* discharge on dialysis*,* reoperation*, or* graft loss*.

## Supplementary Material

The supplemental table shows the detailed descriptive statistics of donor and recipient related covariables of the 25 individuals that were transplanted between 3AM-6AM as well as proportions and distributions of the corresponding data of all patients that were transplanted outside the 3AM-6AM time interval. Normality of distribution of continuous data was analyzed with the Anderson-Darling Test (p < 0.05 indicated a non-normal distribution of data). The proportions of categorial variables and distribution of continuous in- and output data were compared between these two groups using the Fisher's exact Test or the Levene's Test were appropriate. Analyzed were the donor related variables *donor-age*, *BMI*, *Urea*, *Creatinine*, *Sodium(Na+*), *Potassium (K+*), *24h urine production* and *urine production of the last hour* and the recipient related variables *age*, *BMI*, operating time (OT), *cold ischemic time (CIT)*, *anastomosis time (AT)*, *>1 arterial anastomosis [yes/no]*, *ureter stenting [yes/no]*, *1st Surgeons CUSUM*, and the output variates *reoperation[yes/no]*, *perioperative graft loss[yes/no]*, *surgery related perioperative graft loss[yes/no]*, *postoperative dialysis[yes/no]*and *discharge on dialysis[yes/no]*. There were no significant differences between the two groups with respect to the analyzed donor data (p > 0.200, Leven's Test). There were also no significant differences comparing the recipient related variables *age*, *BMI*, *operating time (OT)*, *anastomosis time (AT)*, *>1 arterial anastomosis [yes/no]*, *ureter stenting [yes/no]*, and *1st Surgeons CUSUM*. The only variable which was significantly different was cold ischemic time (CIT), which was shorter in the 3AM-6AM interval group (median=725, mean=903 , SD=542) as compared to the group of patients that were transplanted outside the 3AM-6AM time interval (median=860, mean=917, SD=367) (p=0.004, Levene's Test). The rate of *perioperative graft loss due to surgical reasons* was significantly higher in the 3AM-6AM interval group as compared to the group of patients that were transplanted outside the 3AM-6AM time interval (12% vs. 1.4%, Fisher's Exact Test p=0.007). The 3AM-6AM interval group also had higher rates of *reoperation* (32% vs 17%) and *discharge on dialysis* (25% vs 11%) as compared to the group of patients that were transplanted outside the 3AM-6AM time interval, but the differences were slightly above the significance level of 0.050 (Fisher's Exact Test, p=0.060 and p=0.056, respectively).
The supplemental figure a) shows a X^2^-test comparing the proportions of teaching operations between the 3h day- and night-time intervals. The proportion of teaching operations during the critical hours from 3AM-6AM was neither different to the extrema with lowest proportions of teaching operations (9PM-12AM) nor to the extrema with highest proportion of teaching operations (6AM-9AM) and also not different to any other 3h interval.
Supplemental figure b) shows a comparison of the 3h day- and night-time intervals with respect to the variances of the 1^st^ surgeon's CUSUM. There was no significant differences between any of the compared 3h-time intervals (Leven's test p=0.627).
The supplemental material shows that there was no data disparity or data bias that possibly could have compromised the analyses or the conclusions that were drawn. Moreover, the fact that CIT was significantly shorter (!) in the 3AM-6AM time interval group and that this group was more vulnerable for reoperation und surgery related complications strengthens the conclusion that unnecessarily performed kidney transplantations during high-risk morning hours with the only intention to reduce CIT, but at the expense of surgical quality, might be a bad decision. Especially as prolonged CIT only impacts on the endpoint of delayed graft function, but not on *perioperative graft loss*, *reoperation* and *discharge on dialysis*.

## Figures and Tables

**Table 1:** Univariable binary regression of circadian risk development for binary output variables. Shown are the results of univariable regression analyses of the circadian risk development per 3-hour day and night shift intervals for the investigated endpoints *hospital discharge on dialysis [Yes/No]*, *delayed graft function [Yes/No], *early *postoperative graft loss [Yes/No]*, *postoperative graft loss due to surgical reasons [Yes/No]*, and *reoperation [Yes/No]. *Values of calculated odds ratios of each time interval were plotted on radar-plots to visualize the circadian risk developments of each investigated endpoint.

	3 h interval	No	Yes	%	Univariable binary regression			*χ* ^2^	Circadian risk development
		*n* = 1044	*n* = 218		*p*	OR	95% CI		

Reoperation	12 AM–3 AM (*n* = 96)	81	15	15.6	0.657	0.878	0.496–1.555	0.653	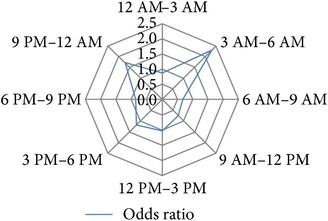
	**3 AM–6 AM **(**n** = 25)	**17**	**8**	**32**	**0.056**	**2.301**	**0.980–5.402**	**0.070**	
	6 AM–9 AM (*n* = 134)	117	17	12.7	0.140	0.670	0.394–1.140	0.124	
	9 AM–12 PM (*n* = 267)	229	38	14.2	0.140	0.751	0.514–1.098	0.131	
	12 AM–3 PM (*n* = 274)	226	48	17.5	0.904	1.022	0.718–1.454	0.904	
	3 PM–6 PM (*n* = 169)	136	33	19.5	0.406	1.191	0.789–1.798	0.412	
	6 PM–9 PM (*n* = 157)	133	24	15.3	0.482	0.847	0.534–1.344	0.475	
	**9 PM–12 AM **(**n** = 140)	**105**	**35**	**25.0**	**0.011**	**1.710**	**1.131–2.588**	**0.014**	

	3 h interval	No	Yes	%	Univariable binary regression			*χ* ^2^	Circadian risk development
		*n* = 1207	*n* = 55		*p*	OR	95% CI		

Perioperative graft loss	12 AM–3 AM (*n* = 96)	94	2	2.1	0.269	0.447	0.107–1.862	0.211	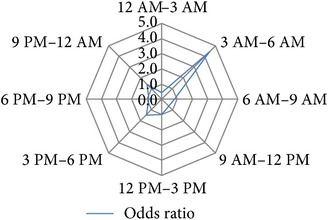
	**3 AM–6 AM **(**n** = 25)	**21**	**4**	**16.0**	**0.008**	**4.430**	**1.467–13.38**	**0.024**	
	6 AM–9 AM (*n* = 134)	129	5	3.7	0.707	0.836	0.327–2.133	0.701	
	9 AM–12 PM (*n* = 267)	259	8	3.0	0.224	0.623	0.291–1.335	0.200	
	12 PM–3 PM (*n* = 274)	262	12	4.4	0.984	1.007	0.523–1.937	0.984	
	3 PM–6 PM (*n* = 169)	159	10	5.9	0.289	1.465	0.724–2.965	0.307	
	6 PM–9 PM (*n* = 157)	151	6	3.8	0.725	0.856	0.361–2.033	0.720	
	9 PM–12 AM (*n* = 140)	132	8	5.7	0.407	1.386	0.641–2.997	0.423	

	3 h interval	No	Yes	%	Univariable binary regression			*χ* ^2^	Circadian risk development
		*n* = 1241	*n* = 21		*p*	OR	95% CI		

Perioperative graft loss due to surgical reasons	12 AM–3 AM (*n* = 96)	95	1	1	0.616	0.597	0.079–4.496	0.588	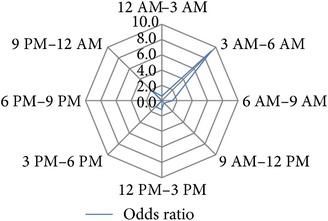
	**3 AM–6 AM **(**n** = 25)	**22**	**3**	**12**	**0.001**	**9.242**	**2.537–33.67**	**0.007**	
	6 AM–9 AM (*n* = 134)	131	3	2.2	0.583	1.413	0.411–4.863	0.598	
	9 AM–12 PM (*n* = 267)	265	2	0.7	0.205	0.388	0.090–1.677	0.151	
	12 PM–3 PM (*n* = 274)	269	5	1.8	0.813	1.130	0.410–3.113	0.815	
	3 PM–6 PM (*n* = 169)	166	3	1.8	0.902	1.080	0.315–3.708	0.903	
	6 PM–9 PM (*n* = 157)	157	0	0	0.996	0.000	0.000	0.018	
	9 PM–12 AM (*n* = 140)	136	4	2.9	0.249	1.913	0.635–5.769	0.281	

	3 h interval	No	Yes	%	Univariable binary regression			*χ* ^2^	Circadian risk development
		*n* = 601	*n* = 261		*p*	OR	95% CI		

Delayed graft function	12 AM–3 AM (*n* = 73)	48	25	34.2	0.441	1.220	0.735–2.026	0.445	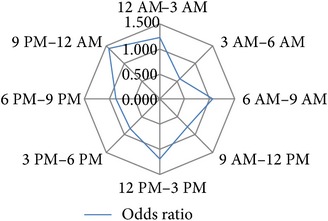
	3 AM–6 AM (*n* = 15)	12	3	20.0	0.388	0.571	0.160–2.040	0.364	
	6 AM–9 AM (*n* = 99)	68	31	31.3	0.812	1.056	0.672–1.660	0.812	
	9 AM–12 PM (*n* = 186)	137	49	26.3	0.188	0.783	0.544–1.127	0.183	
	12 PM–3 PM (*n* = 181)	121	60	33.1	0.345	1.184	0.834–1.681	0.347	
	3 PM–6 PM (*n* = 112)	82	30	26.8	0.389	0.822	0.526–1.284	0.384	
	6 PM–9 PM (*n* = 105)	76	29	27.6	0.527	0.863	0.548–1.361	0.524	
	9 PM–12 AM (*n* = 91)	57	34	37.4	0.121	1.429	0.910–2.246	0.126	

	3 h interval	No	Yes	%	Univariable binary regression			*χ* ^2^	Circadian risk development
		*n* = 862	*n* = 102		*p*	OR	95% CI		

Discharge on dialysis	12 AM–3 AM (*n* = 77)	73	4	5.1	0.119	0.441	0.158–1.233	0.081	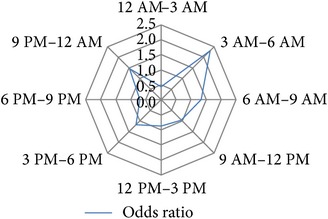
	3 AM–6 AM (*n* = 19)	15	4	21.1	0.145	2.305	0.750–7.082	0.178	
	6 AM–9 AM (*n* = 114)	99	15	13.2	0.342	1.329	0.739–2.389	0.354	
	9 AM–12 PM (*n* = 207)	186	21	10.1	0.818	0.942	0.568–1.564	0.817	
	12 PM–3 PM (*n* = 200)	181	19	9.5	0.577	0.861	0.510–1.456	0.572	
	3 PM–6 PM (*n* = 127)	112	15	11.8	0.629	1.155	0.645–2.068	0.633	
	6 PM–9 PM (*n* = 114)	105	9	7.9	0.323	0.698	0.342–1.425	0.303	
	9 PM–12 AM (*n* = 106)	91	15	14.2	0.208	1.461	0.810–2.634	0.223	

**Table 2:** Proportions of *teaching operations* and distribution of the cumulative sum *(CUSUM)* of the 1st surgeons' kidney transplantations over the 3 h time intervals. Shown are the distribution of teaching operations and the distribution of the cumulative sum *(CUSUM)* of the 1st surgeons' kidney transplantations over the 3 h time intervals. There was no significant difference in distribution of CUSUM between the 3-hour intervals (Levene's test, *p* = 0.627) (Supplemental Figure b). The proportion of teaching operations was unequally distributed between the 9 PM and 12 AM time interval and the two time intervals of 6 AM–9 AM and 9 AM–12 PM, with a significant higher proportion of teaching operations during the morning shift hours between 6 AM and 12 PM (Chi^2^ test *p* < 0.05) (Supplemental Figure a). The proportion of teaching operations within the 3 AM–6 AM interval was not significantly different compared to all other 3-hour intervals (Chi^2^ test *p* > 0.05).

3 h time interval	Teaching operation				CUSUM				
	Expected (*n*)	Counted (*n*)	%	95% CI	Mean	Max	Min	Med	SD
12 AM–3 AM (*n* = 96)	65	62	65	54.16; 74.08	54.5	345	1	37.5	52.3
3 AM–6 AM (*n* = 25)	17	17	68	46.50; 85.05	56.8	235	4	39.0	52.0
6 AM–9 AM (*n* = 134)	90	103	77	68.80; 83.71	52.9	341	1	29.5	60.1
9 AM–12 PM (*n* = 267)	180	194	73	66.89; 77.91	45.0	330	1	29.0	55.5
12 PM–3 PM (*n* = 274)	185	191	70	63.89; 75.09	39.6	361	1	22.0	51.9
3 PM–6 PM (*n* = 169)	114	112	66	58.61; 73.35	39.7	350	1	26.0	46.5
6 PM–9 PM (*n* = 157)	106	96	61	53.05; 68.81	49.8	347	1	33.0	51.9
9 PM–12 AM (*n* = 140)	94	76	54	45.66; 62.72	50.9	346	1	38.0	54.5

**Table 3:** Regression analysis of the risks for *reoperation* (*n* = 218). Shown are the results of univariable and multivariable binary regression analyses to determine the odds ratios of the investigated variables for the risk of *reoperation* (*n* = 218) during primary hospitalization. Analyzed were all 1262 standard kidney transplants into nonpreoperated sites performed between 1 January 2000 and 31 October 2013. Included into risk-adjusted multivariable analyses were those variables with a *p* value < 0.2 in univariable regression analyses.

Continuous variables	Descriptive statistics				Univariable binary regression			Risk-adjusted multivariable binary regression		
	Med	Mean	Range	MV^§^	*p*	OR	95% CI	*p*	OR	95% CI
				*n*/%						
*Donor age [yr.]*	*53*	*52*	*5–88*	*4/0.3*	*0.003*	*1.015*	*1.005–1.025*	*0.013*	*1.013*	*1.003–1.024*
*Donor BMI [kg/m* ^*2*^ *]*	*25*	*25*	*15–38*	*4/0.3*	*0.103*	*1.029*	*0.994–1.065*	*0.038*	*1.040*	*1.002–1.079*
Donor creatinine [*μ*mol/l]	70	91	0–8840	0	0.756	1.000	0.999–1.001	Not selected^1^		
Recipient age [yr.]	55	54	18–77	0	0.802	1.001	0.990–1.013			
Recipient BMI [kg/m^2^]	25	25	15–38	35/2.8	0.521	1.013	0.975–1.052			
CIT [min.]	858	916	125–2458	65/5.2	0.030	1.000	1.000–1.001	0.055	Not calculated^2^	
1st surgeon's CUSUM	29	46	1–361	12/1.0	0.761	1.000	0.998–1.003	Not selected^1^		

Categorical variables			*N*	MV^§^	*p*	OR	95% CI	*p*	OR	95% CI
				*n*/%						

*Night shift surgery 3 AM–6 AM*			*25*	*0*	*0.004*	*3.298*	*1.461–7.443*	*0.006*	*3.335*	*1.421–7.828*
Recipient's right fossa			740	0	0.279	1.180	0.875–1.593	Not selected^1^		
Right donor kidney			611	0	0.062	0.755	0.563–1.014	0.109	Not calculated^2^	
Number of arteries										
One			947	0	Reference			Collinearity with number of arterial anastomoses		
>one			315		0.258	1.208	0.871–1.677			
Numbers of arterial anastomoses										
One			1140	0	Reference			Reference		
>one			122		0.083	1.489	0.950–2.335	0.142	Not calculated^2^	
Number of veins										
One			1220	0	Reference			Not selected^1^		
>one			42		0.258	1.520	0.736–3.141			
Numbers of venous anastomoses										
One			1261	0	Reference					
>one			1		1.000	0.000	0.000			
*Stenting of ureter anastomosis*										
Nonstented			380	5/0.4	Reference			Reference		
*Stented*			*877*		*0.003*	*0.628*	*0.463–0.853*	*0.001*	*0.579*	*0.415–0.809*

^1^Not selected for multivariable regression because of a *p* value > 0.2 in univariable analyses.

^2^Odds ratios and 95% CI were not calculated because of a *p* value > 0.05 in multivariable analyses.

^§^Little's MCAR test: Chi-Square = 31.909, DF = 33, and *p* = 0.521.

**Table 4:** Regression analysis of the risks for *perioperative graft loss* (*n* = 55). Shown are the results of univariable and multivariable binary regression analyses to determine the odds ratios of the investigated variables for the risk of *perioperative graft loss* (*n* = 55) during primary hospitalization period. Analyzed were all 1262 cases with standard kidney transplantations into nonpreoperated sites between 1 January 2000 and 31 October 2013. Included into risk-adjusted multivariable analyses were only transplant variables with a *p* value < 0.2 in univariable regression analyses.

Continuous variables	Descriptive statistics				Univariable binary regression			Risk-adjusted multivariable binary regression		
	Med	Mean	Range	MV^§^	*p*	OR	95% CI	*p*	OR	95% CI
				*n*/%						
*Donor age [yr.]*	*53*	*52*	*5–88*	*4/0.3*	*0.003*	*1.029*	*1.009–1.048*	*0.017*	*1.027*	*1.005–1.050*
Donor BMI [kg/m^2^]	25	25	15–38	4/0.3	0.086	1.051	0.993–1.112	0.276	Not calculated^2^	
Donor creatinine [*μ*mol/l]	70	91	0–8840	0	0.835	1.000	0.998–1.002	Not selected^1^		
Recipient age [yr.]	55	54	18–77	0	0.580	1.006	0.985–1.028			
*Recipient BMI [kg/m* ^*2*^ *]*	*25*	*25*	*15–38*	*35/2.8*	*0.032*	*1.080*	*1.007–1.158*	*0.016*	*1.103*	*1.019–1.194*
CIT [min.]	858	916	125–2458	65/5.2	0.059	1.001	1.000–1.001	0.481	Not calculated^2^	
1st surgeon's CUSUM	29	46	1–361	12/1.0	0.615	1.001	0.997–1.006	Not selected^1^		

Categorical variables			*N*	MV^§^	*p*	OR	95% CI	*p*	OR	95% CI
				*n*/%						

*Night shift surgery 3 AM–6 AM*			*25*	*0*	*0.004*	*5.131*	*1.711–15.389*	*0.003*	*5.543*	*1.758–17.47*
Recipient's right fossa			740	0	0.500	0.844	5.15–1.381	Not selected^1^		
Right donor kidney			611	0	0.715	0.913	0.559–1.489			
Number of arteries										
One			947	0	Reference			Collinearity with number of arterial anastomoses		
>one			315		0.043	1.716	1.017–2.893			
Number of arterial anastomoses										
One			1140	0	Reference			Reference		
>one			122		0.060	1.853	0.974–3.525	0.069	Not calculated^2^	
Number of veins										
One			1220	0	Reference			Not selected^1^		
>one			42		0.963	0.967	0.231–4.053			
Numbers of venous anastomoses										
One			1261	0	Reference					
>one			1		0.999	0.000	0.000			
Stenting of ureter anastomosis										
Nonstented			380	5/0.4	Reference					
Stented			877		0.309	1.348	0.759–2.395			

^1^Not selected because of a *p* value > 0.2 in univariable analyses.

^2^Odds ratios and 95% CI were not calculated because of a *p* value > 0.05 in multivariable analyses.

^§^Little's MCAR test: Chi-Square = 31.909, DF = 33, and *p* = 0.521.

**Table 5:** Regression analysis of the risks of *perioperativegraft loss due to surgical reasons* (*n* = 21). Shown are the results of univariable and multivariable binary regression analyses to determine the odds ratios of the investigated variables for the risk of *perioperativegraft loss due to surgical reasons* (*n* = 21). Analyzed were all 1262 cases with standard kidney transplantations into nonpreoperated sites between 1 January 2000 and 31 October 2013. Included into risk-adjusted multivariable analyses were only transplant variables with a *p* value < 0.2 in univariable regression analyses.

Continuous variables	Descriptive statistics				Univariable binary regression			Risk-adjusted multivariable binary regression		
	Med	Mean	Range	MV^§^	*p*	OR	95% CI	*p*	OR	95% CI
				*n*/%						
Donor age [yr.]	53	52	5–88	4/0.3	0.270	1.016	0.988–1.045	Not selected^1^		
Donor BMI [kg/m^2^]	25	25	15–38	4/0.3	0.466	1.035	0.943–1.136			
Donor creatinine [*μ*mol/l]	70	91	0–8840	0	0.646	0.998	0.992–1.005			
Recipient age [yr.]	55	54	18–77	0	0.823	0.996	0.963–1.030			
*Recipient BMI [kg/m* ^*2*^ *]*	*25*	*25*	*15–38*	*35/2.8*	*0.025*	*1.129*	*1.015–1.265*	*0.019*	*1.139*	*1.021–1.270*
CIT [min.]	858	916	125–2458	65/5.2	0.681	1.00	0.999–1.001	Not selected^1^		
1st surgeon's CUSUM	29	46	1–361	12/1.0	0.350	0.995	0.984–1.006			

Categorical variables			*N*	MV^§^	*p*	OR	95% CI	*p*	OR	95% CI
				*n*/%						

*Night shift surgery 3 AM–6 AM*			*25*	*0*	*0.001*	*9.235*	*2.535–33.647*	*<0.001*	*10.96*	*2.909–41.31*
Recipient's right fossa			740	0	0.558	0.773	0.326–1.833	Not selected^1^		
Right donor kidney			611	0	0.218	1.747	0.719–4.245			
Number of arteries										
One			947	0	Reference					
>one			315		0.375	1.515	0.606–3.787			
*Number of arterial anastomoses*										
One			1140	0	Reference			Reference		
*>one*			*122*		*0.035*	*3.002*	*1.080–8.343*	*0.029*	*3.174*	*1.123–8.973*
Number of veins										
One			1220	0	Reference			Not selected^1^		
>one			42		0.713	1.463	0.192–11.169			
Numbers of venous anastomoses										
One			1261	0	Reference					
>one			1		1.000	0.000	0.000			
Stenting of ureter anastomosis										
Nonstented			380	5/0.4	Reference					
Stented			877		0.609	1.305	0.471–3.617			

^1^Not selected because of a *p* value > 0.2 in univariable analyses.

^§^Little's MCAR test: Chi-Square = 31.909, DF = 33, and *p* = 0.521.

**Table 6:** Regression analysis of the risks for *delayed graft function* (*n* = 272). Shown are the results of univariable and multivariable binary regression analyses to determine the odds ratios of the investigated variables for the risk of *delayed graft function* (*n* = 272). Dialysis data were retrospectively available for 985 cases. Patients with perioperative graft nephrectomy and with discharge on dialysis were censored (*n* = 102). Analyzed were all remaining cases (*n* = 883). Included into risk-adjusted multivariable analyses were only transplant variables with a *p* value ≤ 0.2 in univariable regression analyses.

Continuous variables	Descriptive statistics				Univariable binary regression			Risk-adjusted multivariable binary regression		
	Med	Mean	Range	MV^§^	*p*	OR	95% CI	*p*	OR	95% CI
				*n*/%						
*Donor age [yr.]*	*54*	*53*	*6–88*	*3/0.3*	*<0.001*	*1.018*	*1.008–1.027*	*0.001*	*1.017*	*1.010–1.033*
Donor BMI [kg/m^2^]	26	26	12–52	3/0.3	0.510	1.011	0.978–1.046	Not selected^1^		
Donor creatinine [*μ*mol/l]	68	95	0–8408	0	0.776	1.000	1.000-1.000			
Recipient age [yr.]	56	54	18–77	0	0.013	1.015	1.003–1.027	0.742	Not calculated^2^	
*Recipient BMI [kg/m* ^*2*^ *]*	*25*	*25*	*15–38*	*14/1.6*	*0.001*	*1.068*	*1.029–1.109*	*0.001*	*1.074*	*1.031–1.119*
*CIT [min.]*	*785*	*846*	*125–2286*	*16/1.9*	*<0.001*	*1.002*	*1.001–1.002*	*<0.001*	*1.002*	*1.001–1.002*
1st surgeon's CUSUM	29	46	1–361	5/0.6	0.770	1.000	0.997–1.002	Not selected^1^		

Categorical variables			*N*	MV^§^	*p*	OR	95% CI	*p*	OR	95% CI
				*n*/%						

Night shift surgery 3 AM–6 AM			16	0	0.303	0.515	0.146–1.822	Not selected^1^		
Recipient's right fossa			555	0	0.013	0.689	0.514–0.924	0.065	Not calculated^2^	
Right donor kidney			425	0	0.618	1.076	0.808–1.432	Not selected^1^		
Number of arteries										
One			667	0	Reference					
>one			216		0.432	1.146	0.822–1.599			
Number of arterial anastomoses										
One			802	0	Reference			Reference		
>one			81		0.006	1.927	1.212–3.064	0.087	Not calculated^2^	
Number of veins										
One			852	0	Reference			Not selected^1^		
>one			31		0.835	0.920	0.418–2.025			
Number of venous anastomoses										
One			882	0	Reference					
>one			1		1.000	0.000	0.000			
Stenting of ureter anastomosis										
Nonstented			214	0	Reference					
Stented			669		0.161	0.791	0.571–1.098			

^1^Not selected due to *p* value > 0.2 in univariable analyses.

^2^Odds ratios and 95% CI were not calculated because of a *p* value > 0.05 in multivariable analyses.

^§^Little's MCAR test: Chi-Square = 23.458, DF = 23, and *p* = 0.434.

**Table 7:** Regression analysis of the risks for *hospital discharge on dialysis* (*n* = 102). Shown are the results of univariable and multivariable binary regression analyses to determine the odds ratios of the investigated variables for the risk of *hospital discharge on dialysis* (*n* = 102). Dialysis data were retrospectively available only for the period between 19 May 2003 and 31 Oct 2013. Analyzed were all cases (*n* = 985) with standard kidney transplantations into nonpreoperated sites. Included into risk-adjusted multivariable analyses were only transplant variables with a *p* value ≤ 0.2 in univariable regression analyses.

Continuous variables	Descriptive statistics				Univariable binary regression			Risk-adjusted multivariable binary regression		
	Med	Mean	Range	MV^§^	*p*	OR	95% CI	*p*	OR	95% CI
				*n*/%						
*Donor age [yr.]*	*53*	*52*	*5–88*	*3/0.3*	*<0.001*	*1.028*	*1.013–1.044*	*0.001*	*1.030*	*1.012–1.048*
Donor BMI [kg/m^2^]	25	26	12–52	3/0.3	0.275	1.026	0.980–0.1075	Not selected^1^		
Donor creatinine [*μ*mol/l]	70	91	0–8408	0	0.552	0.999	0.996–1.002			
Recipient age [yr.]	56	54	18–77	0	0.181	1.012	0.995–1.029	0.418	Not calculated^2^	
Recipient BMI [kg/m^2^]	25	25	15–38	33/3.4	0.129	1.041	0.988–1.097	0.178	Not calculated^2^	
CIT [min.]	858	917	125–2458	6/0.6	0.289	1.000	1.000–1.001	Not selected^1^		
1st surgeon's CUSUM	29	46	1–361	17/1.8	0.975	1.000	0.996–1.004			

Categorical variables			*N*	MV^§^	*p*	OR	95% CI	*p*	OR	95% CI
				*n*/%						

Night shift surgery 3 AM–6 AM			20	0	0.163	2.212	0.725–6.747	0.214	Not calculated^2^	
Right recipient's fossa			614	0	0.229	0.775	0.512–1.174	Not selected^1^		
Right donor kidney			474	0	0.986	0.996	0.661–1.502			
Number of arteries										
One			735	0	Reference			Collinearity with number of arterial anastomoses		
>one			250		0.053	1.544	0.995–2.396			
Number of arterial anastomoses										
One			888	0	Reference			Reference		
>one			97		0.039	1.842	1.031–3.292	0.066	Not calculated^2^	
Number of veins										
One			950	0	Reference			Not selected^1^		
>one			35		0.832	1.122	0.388–3.245			
Number of venous anastomosis										
One			984	0	Reference					
>one			1		1.000	0.000	0.000			
Stenting of ureter anastomosis										
Nonstented			242	3/0.3	Reference					
Stented			740		0.376	0.811	0.510–1.290			

^1^Not selected because of a *p* value > 0.2 in univariable analyses.

^2^Odds ratios and 95% CI were not calculated because of a *p* value > 0.05 in multivariable analyses.

^§^Little's MCAR test: Chi-Quadrat = 26.171, DF = 33, and *p* = 0.795.

**Table 8:** Literature about CIT-impact on kidney transplantation outcome.

Authors	Year	Endpoint	Number of CIT intervals	CIT interval details	Resolution [hours]	OR calculation method [stepwise forward/blockwise two-sided]
Debout et al. [18]	2015	Graft failure, death	4	6–16 h, 16–24 h, 24–36 h, >36 h	8 and 12	Blockwise two-sided

Gill et al. [19]	2014	DGF	7	0–6 h, 6–12 h, 12–18 h, 18–24 h, 24–30 h, 30–36 h, >36 h	6	Blockwise two-sided

Sert et al. [20]	2014	DGF	3	0–10 h, 10–20 h, 20–30 h, >30 h	10	Blockwise two-sided

van der Vliet et al. [21]	2011	DGF, 5 yr graft survival	5	0–16 h, 16–20 h, 21–25 h, 26–30 h, >30 h	4 and 16	Blockwise two-sided

Quiroga et al. [22]	2006	DGF, AR	5	5–17 h, 18–20 h, 21–24 h, 25–31 h, >32 h	3, 4, 5, 7, 13	Blockwise two-sided

Su et al. [23]	2004	Graft failure	6	0–8 h, 9–16 h, 17–24 h, 25–36 h, 37–48 h, >48 h	8 and 12	Blockwise two-sided

Opelz [24]	2004	Graft failure	5	0–6 h, 7–12 h, 13–24 h, 25–36 h, >36 h	6 and 12	Blockwise two-sided

Smits et al. [25]	2000	Graft failure	4	0–18 h, 19–24 h, 25–36 h, >37 h	5 and 18	Blockwise two-sided

Ojo et al. [26]	1997	DGF	4	0–12 h, 13–24 h, 25–36 h, >36 h	12	Blockwise two-sided

## Abbreviations

AR: Acute rejection
CI: Confidence interval
CIT: Cold ischemic time
CUSUM: Cumulative summation
DGF: Delayed graft function
INF: Initial nonfunction
OR: Odds ratio
*R*^2^: Residual sum of squares
RO: Reoperation.
