# Supplementary material for: Risk Balancing of Cold Ischemic Time against Night Shift Surgery Possibly Reduces Rates of Reoperation and Perioperative Graft Loss
Source: J Transplant. 2017 Jan 19;2017:5362704. doi: 10.1155/2017/5362704 (PMC5288530; doi:10.1155/2017/5362704)
Supplement: Supplementary file 1 — The supplemental table shows the detailed descriptive statistics of donor and recipient related covariables of the 25 individuals that were transplanted between 3AM-6AM as well as proportions and distributions of the corresponding data of all patients that were transplanted outside the 3AM-6AM time interval. Normality of distribution of continuous data was analyzed with the Anderson-Darling Test (p < 0.05 indicated a non-normal distribution of data). The proportions of categorial variables and distribution of continuous in- and output data were compared between these two groups using the Fisher's exact Test or the Levene's Test were appropriate. Analyzed were the donor related variables donor-age, BMI, Urea, Creatinine, Sodium(Na+), Potassium (K+), 24h urine production and urine production of the last hour and the recipient related variables age, BMI, operating time (OT), cold ischemic time (CIT), anastomosis time (AT), >1 arterial anastomosis [yes/no], ureter stenting [yes/no], 1st Surgeons CUSUM, and the output variates reoperation[yes/no], perioperative graft loss[yes/no], surgery related perioperative graft loss[yes/no], postoperative dialysis[yes/no]and discharge on dialysis[yes/no]. There were no significant differences between the two groups with respect to the analyzed donor data (p > 0.200, Leven's Test). There were also no significant differences comparing the recipient related variables age, BMI, operating time (OT), anastomosis time (AT), >1 arterial anastomosis [yes/no], ureter stenting [yes/no], and 1st Surgeons CUSUM. The only variable which was significantly different was cold ischemic time (CIT), which was shorter in the 3AM-6AM interval group (median=725, mean=903 , SD=542) as compared to the group of patients that were transplanted outside the 3AM-6AM time interval (median=860, mean=917, SD=367) (p=0.004, Levene's Test). The rate of perioperative graft loss due to surgical reasons was significantly higher in the 3AM-6AM interval group as compared [file 5362704.f1.pdf]

Supplemental Figure

a

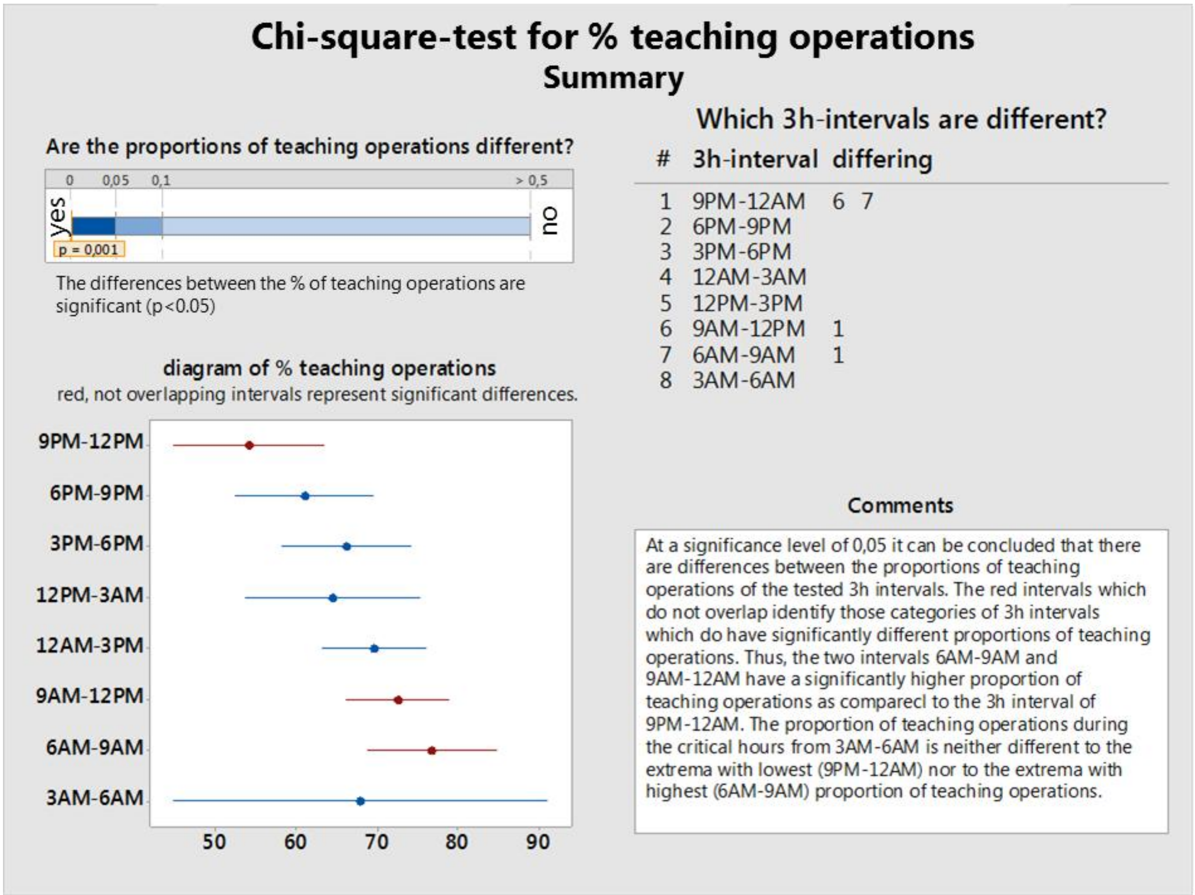

b

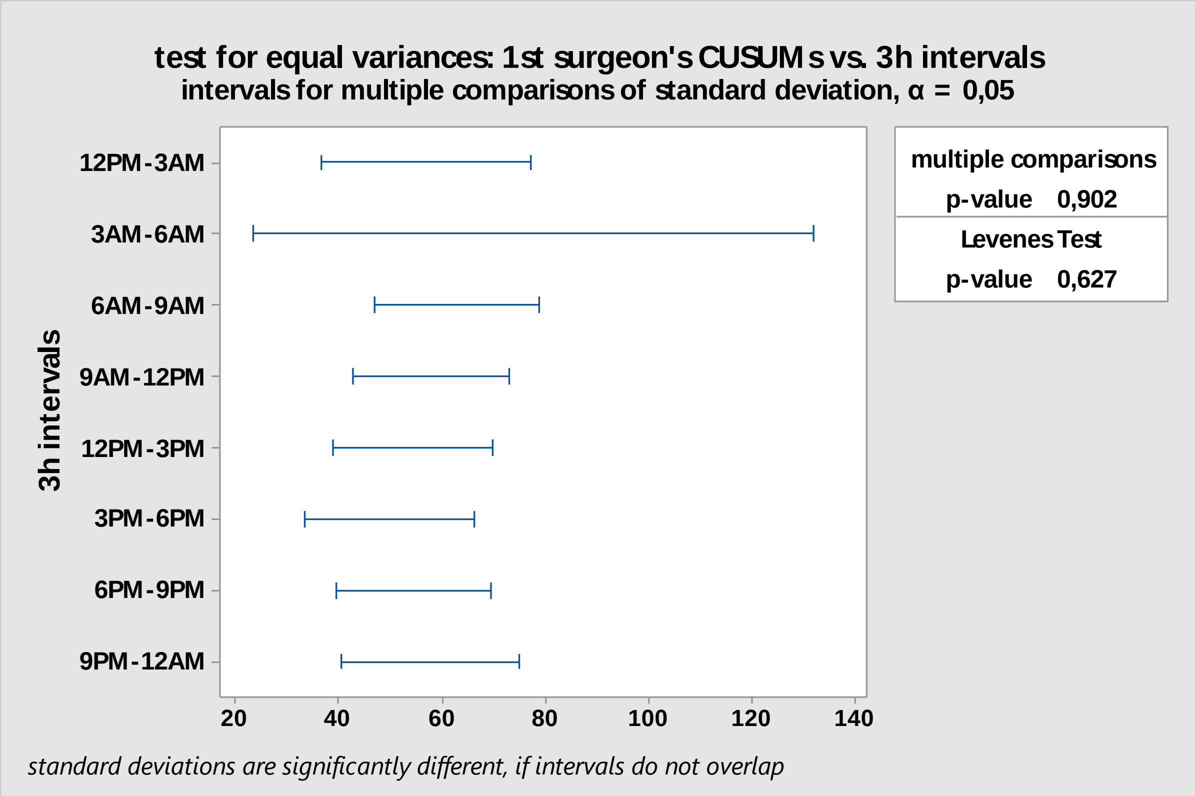

Supplemental Table: Detailed donor and recipient related in- and output data of the 25 individuals transplanted between 3AM-6AM in comparison to patients transplanted outside the 3AM-6AM time interval.

	Pat. #	DONOR CRITERIA								RECIPIENT CRITERIA											
		age [yrs.]	BMI	Urea mmol /l	Crea µmol /l	Na^+ mmol /l	K^+ mmol /l	urine /24h [ml]	urine last hour [ml]	age [yrs.]	BMI	OT [min.]	CIT [min.]	AT [min.]	> 1 art. Anast.	ureter stent	1st Surg. Cusum	reoperation due to …	perioperative graft loss surgery related	postoperative dialysis	discharge on dialysis
inside 3AM - 6AM (n=25)	1	56	25	3,1	146	160	4,8	4740	22	63	19	130	*NV*	*NV*	no	no	4	ureter necr.	-	yes	yes
	2	41	26	3,9	66	159	3,3	6500	500	41	26	130	2170	*NV*	no	no	*NV*	-	-	yes	-
	3	35	25	4,1	74	145	4,2	5000	250	55	21	55	1150	*NV*	no	no	11	bleeding	-	-	-
	4	37	24	*NV*	106	149	3,6	2800	40	61	26	95	1365	28	no	yes	50	-	-	yes	-
	5	48	21	5,4	118	162	4,2	5200	1100	61	28	119	1512	29	no	no	35	-	-	-	-
	6	7	15	15	62	147	3,6	1500	50	65	29	115	1674	25	no	yes	93	-	-	-	-
	7	55	23	7,5	66	136	3,5	*NV*	*NV*	71	20	105	1863	30	no	no	21	-	-	-	-
	8	61	26	6	133	136	3	*NV*	50	65	28	65	948	21	no	no	29	bleeding	-	yes	-
	9	61	26	6	133	136	3	*NV*	50	58	28	75	1132	27	no	yes	28	art. diss.	yes	yes	yes
	10	65	33	2,7	71	158	4,3	2700	120	65	23	146	695	35	no	yes	20	-	-	-	-
	11	17	25	4,5	5	130	4	2860	60	37	22	64	743	17	no	yes	235	-	-	-	-
	12	69	23	5,3	78	142	4,3	2560	50	70	23	109	396	26	no	yes	70	-	-	-	-
	13	76	24	4,4	58	135	4	2000	50	41	24	96	676	24	no	yes	47	-	-	-	-
	14	15	21	2,1	66	158	3,6	3370	100	42	20	105	341	31	no	yes	14	-	-	-	-
	15	68	25	3,7	83	146	3,9	5660	150	68	30	140	352	26	no	yes	26	-	-	-	-
	16	50	27	5,3	55	132	3,9	2500	100	55	25	180	1752	94	yes	yes	17	-	-	-	-
	17	59	24	6	78	177	3,9	5500	500	55	31	160	525	22	no	yes	118	INF	no	yes	yes
	18	55	23	2,7	44	146	4,6	4600	320	41	25	102	314	24	no	yes	16	colon perf.	yes	yes	yes
	19	64	27	3,8	147	145	6,1	6200	400	52	30	75	447	27	no	yes	43	-	-	-	-
	20	75	24	4,3	53	154	5,5	3960	220	70	21	94	618	34	no	yes	116	-	-	-	-
	21	77	29	7,7	106	146	4,3	3100	100	65	30	320	854	27	no	yes	96	ven. thromb.	yes	yes	yes
	22	72	26	6,8	56	143	4,2	4924	200	69	24	97	306	24	no	yes	100	-	-	-	-
	23	63	37	8,2	154	150	3,8	3800	120	56	30	220	707	48	no	yes	89	wound dehisc.	-	yes	-
	24	61	29	8,2	180	147	4,5	5640	400	51	30	116	785	39	no	yes	21	-	-	yes	-
	25	65	24	3,3	50	161	3,5	3700	100	72	23	99	343	42	yes	yes	65	-	-	-	-
	**Mean**	54,1	25,3	5,4	87,5	149	4,1	3982	210,5	58	25,4	120	902,8	31,8	-	-	56,8	-	-	-	-
	**Median**	61	24,8	4,9	74,0	147	4,0	3800	110	61	25	105	725	27	-	-	39	-	-	-	-
	**SD**	18,7	4,0	2,6	41,2	9,7	0,7	1403	235,3	11	3,7	54,8	541,7	15,3	-	-	52	-	-	-	-
	**%**	-	-	-	-	-	-	-	-	-	-	-	-	-	8	76	-	32	12	50	25
	**Anderson-Darling**	0.006	0.005	0.208	0.005	0.337	0.902	0.297	0.005	0.063	0.161	0.005	0.018	0.005	-	-	0.182	-	-	-	-
outside 3AM - 6AM (n=1237)	**Mean**	52	25,9	7,9	106	148	4,3	4330	191	54	25	130	917	36	-	-	46	-	-	-	-
	**Median**	53	25,4	5,3	77	147	4,1	3800	150	55	25	127	860	35	-	-	28	-	-	-	-
	**SD**	16,2	4,1	14,5	297	9,5	4,9	3235	220	13	3,8	35	367	12,5	-	-	53,4	-	-	-	-
	**%**	-	-	-	-	-	-	-	-	-	-	-	-	-	10	70	-	17	1.4	35	11
	**Anderson-Darling**	0,005	0,005	0,005	0,005	0,005	0,005	0,005	0,005	0,005	0,005	0,005	0,005	0,005	-	-	<0.005	-	-	-	-
**Levens's Test**		0,734	0,713	0,277	0,735	0,601	0,83	0,398	0,263	0,239	0,840	0,104	0,004	0,452	-	-	0.945	-	-	-	-
**Fisher's Test**		-	-	-	-	-	-	-	-	-	-	-	-	-	1.0	0.661	-	0.060	0.007	0.237	0,056

Legend to supplemental table:

Shown are the detailed data of donor and recipient related covariables of the 25 individuals that were transplanted between 3AM-6AM as well as proportions and distributions of the corresponding data of all patients that were transplanted outside the 3AM-6AM time interval. Normality of distribution of continuous data was analyzed with the Anderson-Darling Test ($p < 0.05$ indicated a non-normal distribution of data). The proportions of categorial variables and distribution of continuous in- and output data were compared between the two groups using the Fisher's exact Test or the Levene's Test were appropriate.

CIT was significantly shorter in the 3AM-6AM interval group (mean=903, median=725, SD=542) as compared to the group of patients that were transplanted outside the 3AM-6AM time interval (mean=917, median=860, SD=367) (p=0.004, Levene's Test). The rate of *perioperative graft loss due to surgical reasons* was significantly higher in the 3AM-6AM interval group as compared to the group of patients that were transplanted outside the 3AM-6AM time interval (12% vs. 1.4%, Fisher's Exact Test p=0.007). The 3AM-6AM interval group also had higher rates of *reoperation* (32% vs 17%) and *discharge on dialysis* (25% vs 11%) as compared to the group of patients that were transplanted outside the 3AM-6AM time interval, but the differences were slightly above the significance level of .05 (Fisher's Exact Test, p=0.060 and p=0.056, respectively).
